# Supplementary material for: Content-rich biological network constructed by mining PubMed abstracts
Source: BMC Bioinformatics. 2004 Oct 8;5:147. doi: 10.1186/1471-2105-5-147 (PMC528731; doi:10.1186/1471-2105-5-147)
Supplement: Additional File 2 — The original results of the above study (non-essential files are deleted to keep the file size under the limit set by BMC bioinformatics). [file 1471-2105-5-147-S2.bz2 › chilibotAdditionalFile2/dip30/573ID2164219E10940/html/CD28_CD80.html]

 


 **CD28** and **CD80** 
  
Found 551 abstracts in PubMed, retrieved 30.  
 

 What does Google say? 
 PDF only 
| .edu only 

---

**Interactive relationship** (e.g. stimulation, inhibition, etc)

**Stimulatory relationship**- **CD28**  binds to its ligand  **CD80**  or CD86 and transduces the most important costimulatory signal.  Ref: 12636255 Microbiol Immunol, 2003
- CD4 T cell activation is positively  **CD28**  and negatively CTLA 4 regulated by the costimulatory ligands  **CD80**  and CD86.  Ref: 12496387 J Immunol, 2003
- Many new vaccines will be based upon soluble recombinant antigens, and in vaccination with these antigens  **CD80**  and CD86 would normally be expressed on activated antigen presenting cells and additional stimulation through  **CD28**  would not be predicted to enhance responses further.  Ref: 12594842 Eur J Immunol, 2003
- Using chimeric rat mouse  **CD28**  molecules, we show that the superagonists bind exclusively to the laterally exposed C D loop of the immunoglobulin like domain of  **CD28**  whereas conventional, costimulatory mAbs recognize an epitope close to the binding site for the natural  **CD80**  CD86 ligands.  Ref: 12707299 J Exp Med, 2003
**Neutral relationship**- The importance of interactions of  **CD80**  CD86 and  **CD28**  in activating CD4 T cells was clear.  Ref: 12794123 J Immunol, 2003
- Condensed aromatic peptide family of microbial metabolites, inhibitors of  **CD28**   **CD80**  interactions.  Ref: 12565974 Bioorg Med Chem Lett, 2003
- An effective humoral response requires bidirectional interactions between B cells and CD4 T cells, critical of which are interactions between  **CD80**  CD86 expressed on activated B cells and  **CD28**  expressed on responder CD4 T cells.  Ref: 12794123 J Immunol, 2003
- The  **CD28**  independence of effector T cells within germinal centers suggested that a key function for  **CD80**  CD86 under these circumstances might be to provide negative regulatory signals via the  **CD28**  homologue CTLA 4.  Ref: 12496387 J Immunol, 2003
- Fas ligand FasL, that has the inherent capacities to tether the T cell inhibitor FasL CD95 ligand to the surfaces of B7  **CD80**  and CD86 positive APC via CTLA 4 B7 interaction, and in so doing, to simultaneously interfere with B7 to  **CD28**  T cell activation signals.  Ref: 12794109 J Immunol, 2003
- BACKGROUND  **CD80**  and CD86, cell surface molecules found only on antigen presenting cells APCs, are required for activation of CD4 postitve CD4 T cells by interaction with  **CD28**  cytotoxic T lymphocyte associated antigen 4 on T cells.  Ref: 12722029 Am J Kidney Dis, 2003
- The CD86 major histocompatibility complex class II MHC II cells and  **CD28**  CD4 T cells were decreased by SST treatment, while  **CD80**  MHC II cells, CD40 MHC II cells and CD154 CD4 T cells showed no change.  Ref: 12501009 Jpn J Pharmacol, 2002
- Attempts at blocking the  **CD28**  B7s  **CD80**  CD86 pathway are currently underway with the receptor fusion protein, LEA29Y a second generation CTL4Aig, and humanized mAbs to CD 80  [ **CD80** ]  and CD86.  Ref: 12773967 Minerva Urol Nefrol, 2003

**Non-interactive relationship** (e.g. studied together, co-existance, homology, etc.)

- These results demonstrate, for the first time, a critical contribution of the  **CD28**   **CD80**  CD86 costimulatory pathway not only to natural protection against primary T. cruzi infection but also to DNA vaccine induced protective immunity to Chagas disease.  Ref: 12761091 Infect Immun< MedlineTA>Infect Immun, 2003
- The  **CD80**  86  **CD28**  and CD40 CD40 ligand costimulatory pathways are essential for Th cell dependent B cell responses that generate high affinity, class switched Ab in vivo.  Ref: 12517941 J Immunol, 2003
- The  **CD28**   **CD80**  CD86 mediated T cell costimulatory pathway has been variably implicated in infectious immunity.  Ref: 12761091 Infect Immun< MedlineTA>Infect Immun, 2003
- the contribution of  **CD28**  B7 costimulation to allergen induced release of TLCA was determined through use of CTLA4 Ig fusion protein and neutralizing monoclonal antibodies to  **CD80**  B7.1 and CD86 B7.2.  Ref: 12464958 J Allergy Clin Immunol, 2002
- Expression of CD4, CD8alpha, CD40, CD86,  **CD28** , CD31 and vascular endothelial growth factor was not different between the two tumor groups but expression of CD40 ligand,  **CD80** , NK1.1 and CXCR3 was relatively lower in A11 Mig tumors.  Ref: 12792744 Oncol Rep< MedlineTA>Oncol Rep, 2002
- **CD80** , CD86,  **CD28**  and Cytotoxic T lymphocyte antigen 4 CTLA 4 are well known co stimulatory molecules that form the major co stimulatory pathway essential for full activation of T cells.  Ref: 12679556 J Vet Med Sci, 2003
- the ablation of a normal response following the addition of neutralizing anti CD86  **CD80**  Abs mirrored the response of CD4 T cells to B cells of HIV viremic patients, while the addition of exogenous  **CD28**  ligands partially restored the poor CD4 T cell response to the B cells of HIV viremic patients.  Ref: 12794123 J Immunol, 2003
- In this study, we investigated the role of this costimulatory pathway in resistance to Trypanosoma cruzi infection by using  **CD28**  deficient mice and blocking antibodies against  **CD80**  and CD86.  Ref: 12761091 Infect Immun< MedlineTA>Infect Immun, 2003
- In this study, we report the construction of a monovalent single chain Fv antibody fragment from a high affinity anti human  **CD28**  antibody  **CD28** .3 that blocked adhesion of T cells to cells expressing the  **CD28**  receptor  **CD80** .  Ref: 12649149 Blood< MedlineTA>Blood, 2003
- **CD28**  is an important costimulatory molecule for T cells, and it has been shown that cell surface expression of its ligands,  **CD80**  and CD86, can enhance cellular immune responses against tumor cells these tumor cells do not normally express the ligands.  Ref: 12594842 Eur J Immunol, 2003
- The result of the present study indicated the potential role of the  **CD28**   **CD80**  co stimulation pathway in pathogenesis of allergic diseases in dogs.  Ref: 12679556 J Vet Med Sci, 2003
- Melanoma patients with thinner tumors and those surviving revealed an increase of CD19  **CD80**  and CD19  **CD80**  CD86 cells, as well as a higher concentration of CD3 CD4  **CD28**  cells.  Ref: 12878358 Hum ImmunolHum Immunol, 2003
- Thus, while disrupting either the  **CD80**  86  **CD28**  or the CD40 CD40 ligand costimulatory pathway abrogates T dependent B cell immune responses, the two pathways are nonredundant and mediated by distinct mechanisms.  Ref: 12517941 J Immunol, 2003
- We determined the concentration of peripheral blood lymphocytes, which expressed the  **CD80**  CD86 or  **CD28**  CTLA 4 molecules, in 38 patients with cutaneous melanoma and 27 controls.  Ref: 12878358 Hum ImmunolHum Immunol, 2003
- Antibodies to the  **CD28**  counter ligands  **CD80**  and CD86 inhibited allergen induced release of TLCA in mild asthmatic explants by 94% P < ;.  Ref: 12464958 J Allergy Clin Immunol, 2002
- High expression of  **CD28** ,  **CD80** , CD86, CD40, CTLA4, CD44v variants, and FasL occurred in alopecia areata resistant mouse spleens.  Ref: 12485450 J Invest Dermatol, 2002
- Critical Contribution of  **CD28**   **CD80**  CD86 Costimulatory Pathway to Protection from Trypanosoma cruzi Infection.  Ref: 12761091 Infect Immun< MedlineTA>Infect Immun, 2002
- These include the costimulatory ligand receptor pairs, such as CD40 CD154 and  **CD80**  CD86  **CD28** , CD52 and CD45.  Ref: 12517277 Expert Opin Biol Ther, 2002
- Changes in the number of  **CD80** , CD86, and  **CD28**  peripheral blood lymphocytes have prognostic value in melanoma patients.  Ref: 12878358 Hum ImmunolHum Immunol, 2002
- We hypothesized that in relapsing remitting MS RRMS with or without of IFNbeta treatment and in secondary progressive patients SPMS IL12 and costimulatory molecules  **CD80**  B7 1, CD86 B7 2,  **CD28** , CD40, CD40L would be differentially produced or expressed by MO or T cells.  Ref: 12672403 Clin Immunol, 2003
- Subsequent CTL induction by activated DC requires  **CD80**  CD86 on the DC to interact with the  **CD28**  costimulatory receptor on CD8 T cells.  Ref: 12445291 Immunol Rev, 2002
- Additional phenotyping of these clones showed them to express CD25,  **CD28** ,  **CD80**  and 86 but not CD56 or 57.  Ref: 12709539 Rheumatology Oxford , 2003
- Expression of costimulatory molecules, CD49D and  **CD28**  on T cells and  **CD80**  and CD86 on monocytes, was unchanged.  Ref: 12753529 Respirology< MedlineTA>Respirology, 2003
- All these compounds exhibited good activity against  **CD28**   **CD80**  binding with an IC 50 of 0.4.38 and 0.22 microM, respectively.  Ref: 12565974 Bioorg Med Chem Lett, 2003
